# Quasi-Irreversible Inhibition of CYP2D6 by Berberine

**DOI:** 10.3390/pharmaceutics12100916

**Published:** 2020-09-24

**Authors:** Ha Gyeong Kim, Han Sol Lee, Jang Su Jeon, Young Jae Choi, Yeon Jung Choi, So-Yeol Yoo, Eun-yeong Kim, Kiho Lee, InWha Park, MinKyun Na, Han-Jin Park, Seung-Woo Cho, Jong-Hoon Kim, Jae-Young Lee, Sang Kyum Kim

**Affiliations:** 1 College of Pharmacy, Chungnam National University, Daejeon 34134, Korea; kkimhan403@o.cnu.ac.kr (H.G.K.); sol4273@cnu.ac.kr (H.S.L.); navisu@cnu.ac.kr (J.S.J.); youngjae.choi@kitox.re.kr (Y.J.C.); yjchoi3229@korea.kr (Y.J.C.); nature4857@cnu.ac.kr (S.-Y.Y.); inwha129@kist.re.kr (I.P.); mkna@cnu.ac.kr (M.N.); 2 College of Pharmacy, Korea University, Sejong 30019, Korea; eunyeongkim@korea.ac.kr (E.-y.K.); kiholee@korea.ac.kr (K.L.); 3 Natural Product Informatics Research Center, Korea Institute of Science and Technology (KIST) Gangneung Institute, Gangneung 25451, Korea; 4 Predictive Model Research Center, Korea Institute of Toxicology, Daejeon 34114, Korea; hjpark@kitox.re.kr; 5 Department of Biotechnology, Yonsei University, Seoul 03722, Korea; seungwoocho@yonsei.ac.kr; 6 Laboratory of Stem Cells and Tissue Regeneration, Department of Biotechnology, College of Life Sciences and Biotechnology, Science Campus, Korea University, Seoul 02841, Korea; jhkim@korea.ac.kr

**Keywords:** berberine, cytochrome P450, herb-drug interaction, metabolite identification, drug metabolism

## Abstract

In our previous study, Hwang-Ryun-Hae-Dok-Tang, which contains berberine (BBR) as a main active ingredient, inhibited cytochrome P450 (CYP) 2D6 in a quasi-irreversible manner. However, no information is available on the detailed mechanism of BBR-induced CYP2D6 inhibition. Thus, the present study aimed to characterize the inhibition mode and kinetics of BBR and its analogues against CYP2D6 using pooled human liver microsomes (HLM). BBR exhibited selective quasi-irreversible inhibition of CYP2D6 with inactivation rate constant (*k_inact_*) of 0.025 min^−1^, inhibition constant (*K_I_*) of 4.29 µM, and *k_inact_*/*K_I_* of 5.83 mL/min/µmol. In pooled HLM, BBR was metabolized to thalifendine (TFD), demethyleneberberine (DMB), M1 (proposed as demethylene-TFD), and to a lesser extent berberrubine (BRB), showing moderate metabolic stability with a half-life of 35.4 min and a microsomal intrinsic clearance of 7.82 µL/min/mg protein. However, unlike BBR, those metabolites (i.e., TFD, DMB, and BRB) were neither selective nor potent inhibitors of CYP2D6, based on comparison of half-maximal inhibitory concentration (IC_50_). Notably, TFD, but not DMB, exhibited metabolism-dependent CYP2D6 inhibition as in the case of BBR, which suggests that methylenedioxybenzene moiety of BBR may play a critical role in the quasi-irreversible inhibition. Moreover, the metabolic clearance of nebivolol (β-blocker; CYP2D6 substrate) was reduced in the presence of BBR. The present results warrant further evaluation of BBR–drug interactions in clinical situations.

## 1. Introduction

Herbal medicine often changes the disposition of concomitant drugs by inducing or inhibiting drug-metabolizing enzymes [1,2]. In particular, the herb-drug interaction (HDI) involving the inhibition of major cytochrome P450 (CYP) isozymes (i.e., 1A2, 2A6, 2B6, 2C8, 2C9, 2C19, 2D6, 2E1, and 3A4/5) may increase the drug concentration to a toxic level [3,4,5], causing unexpected adverse effects [6]. This HDI results from not only the herbal medicine itself but also its metabolites, which is called metabolism-dependent inhibition (MDI). Moreover, some metabolites can inhibit CYPs in a quasi-irreversible or irreversible manner, which is attributed to their increased reactivity after metabolism [7,8,9].

Berberine (BBR) is an isoquinoline alkaloid isolated from goldthread (*Coptis chinensis*), goldenseal (*Hydrastis canadensis*), barberry (*Berberis vulgaris*), and Oregon grape (*Berberis aquifolium*) [10,11]. Given its various pharmacological activities, such as anti-diarrheic [12,13], anti-microbial [14], anti-arrhythmic [15,16], anti-cancer [17,18,19,20], anti-inflammatory [21,22], and anti-viral effects [23], clinical studies have been conducted in patients with hyperlipidemia or type 2 diabetes [24,25]. In addition, the pharmacokinetic (PK) properties of BBR have been extensively investigated, including its metabolism. According to a PK study performed in rats, BBR is biotransformed into four major metabolites by CYPs, including berberrubine (BRB), thalifendine (TFD), demethyleneberberine (DMB), and jatrorrhizine (JTZ) [26]. An in vitro study also demonstrated the metabolism of BBR into TFD and DMB by CYP1A2, 2D6, and 3A4 isozymes using human liver microsomes (HLM) and recombinant human CYPs [27].

Similarly, our previous study using HLM showed that BBR and Hwang-Ryun-Hae-Dok-Tang (H-Tang; a BBR-containing traditional herbal formula) inhibit CYP2D6 with half-maximal inhibitory concentration (IC_50_) values of approximately 7.9 µg/mL and 25.0 µg/mL, respectively [28]. Notably, the CYP2D6 inhibition by H-Tang exhibited a quasi-irreversible property, implying that BBR could be associated with the possible formation of a metabolite-intermediate complex (MIC). Thus, herein, we thoroughly characterized the inhibition mechanism of BBR and its analogues against CYP enzymes, especially 2D6. The MDI of CYP2D6 was investigated via the pre-incubation of HLM with BBR or its metabolites, of which kinetic parameters were also determined. The quasi-irreversible MIC formation between BBR and CYP2D6 was demonstrated by assessing the reversibility of enzyme activity using ultracentrifugation coupled with CYP oxidation. Moreover, the potential of HDI between BBR and nebivolol on microsomal metabolism was elucidated in HLM for the first time.

## 2. Materials and Methods

### 2.1. Chemicals and Reagents

BBR, phenacetin, coumarin, bupropion, tolbutamide, dextromethorphan, chlorzoxazone, testosterone (TST), ketoconazole, verapamil, diltiazem, mifepristone, carbamazepine (CBZ), potassium ferricyanide (K_3_Fe(CN)_6_), glucose 6-phosphate (G6P), glucose 6-phosphate dehydrogenase (G6PDH), β-nicotinamide adenine dinucleotide phosphate hydrate (NADP^+^; oxidized form), nebivolol hydrochloride, formic acid were purchased from Sigma-Aldrich (St. Louis, MO, USA). Midazolam (MDZ) was bought from Bukwang Pharmaceutical Co. (Seoul, Korea). *S*-Mephenytoin was purchased from BD Gentest Co. (Woburn, MA, USA). BRB chloride, JTZ, tetrahydroberberine (THB; canadine), and β-nicotinamide adenine dinucleotide phosphate (NADPH; reduced form) were supplied by Toronto Research Chemicals Inc. (Toronto, ON, Canada). DMB and TFD chloride were purchased from Inter Pharm (Goyang, Korea) and WuXi AppTec Co. (Shanhai, China), respectively. Tetrahydroberberrubine·acetate (TRB; purity > 95%) was isolated from the fruits of *Nandina domestica* (see Supplementary Materials). Pooled HLM and recombinant human CYP2D6 (rhCYP2D6; product number: 456217) were obtained from Corning Gentest (Corning, NY, USA) and stored at −80 °C before use. All the other chemicals and reagents used were of analytical grade.

### 2.2. Direct CYP Inhibition Assay Using Pooled HLM

The CYP inhibition assay was conducted as described previously [29,30]. In brief, BBR or its analogue (THB, TRB, BRB, TFD, DMB, or JTZ) at various concentrations (up to 100 µM) was pre-incubated with pooled HLM (0.2 mg/mL) and two different CYP isoform-selective substrate cocktail sets (set A: 50 µM of phenacetin, 5 µM of coumarin, 0.2 µM of amodiaquine, 100 µM of *S*-mephenytoin, 5 µM of dextromethorphan, and 5 µM of MDZ; set B: 50 µM of bupropion, 100 µM of tolbutamide, 50 µM of chlorzoxazone, and 50 µM of TST) in phosphate buffer (0.1 M; pH 7.4) at 37 °C for 5 min. The enzyme reactions were initiated by adding NADPH (1 mM), and the mixtures were incubated at 37 °C for 5 min in a shaking water bath (100 rpm). The reaction was terminated by adding ice-cold acetonitrile (ACN) containing CBZ (100 nM) and 4-methyl-umbelliferone (300 nM) as internal standards (IS). The mixtures were centrifuged, and the supernatants were analyzed using liquid chromatography-tandem mass spectrometry (LC-MS/MS), by which the inhibition of metabolite formation was monitored in the presence of BBR or THB.

The chromatographic separation was performed on an Agilent 1200 series HPLC system (Agilent Technologies, Santa Clara, CA, USA) equipped with an Atlantis^TM^ dC18 column (2.1 × 50 mm, 3 µm; Waters Corporation, Milford, MA, USA) with a SecuityGuard^TM^ C18 guard column (2.0 × 4.0 mm; Phenomenex, Torrance, CA, USA). The mobile phases consisted of double-deionized water (DDW) containing 0.1% (*v*/*v*) of formic acid (A) and ACN containing 0.1% (*v*/*v*) formic acid (B). A linear gradient elution program was used at a flow rate of 0.4 mL/min, where the percentage of mobile phase B according to the time was as follows: 0% at 0–1 min, 45% at 1.1 min, 50% at 4 min, 95% at 4.1–6 min, and 100% at 6.01–7 min, followed by re-equilibration for 2 min. The sample injection volume was 10 µL, and the total run time was 9.0 min. The column and auto-sampler compartments were maintained at 30 °C and 10 °C, respectively, throughout the analysis. The detection was performed using an API4000 Q-TRAP system equipped with a Turbo V Ion Spray^TM^ source (Applied Biosystems, Foster City, CA, USA). The operating conditions were optimized as follows: ionization voltage: 5500 V for positive-ion mode and −4500 V for negative-ion mode; ion source temperature: 550 °C; nebulizing gas flow: 50 L/min; auxiliary gas flow: 40 L/min; and curtain gas flow: 30 L/min. The samples were analyzed via multiple reaction monitoring (MRM) mode. More detailed MRM parameters of analytes are listed in Supplementary Materials (Table S1). The data acquisition and processing were performed using Analyst^TM^ software (version 1.5.2; Applied Biosystems, Foster City, CA, USA).

### 2.3. In Vitro Metabolism-Dependent CYP2D6 Inhibition Assay Using Pooled HLM and rhCYP2D6

Pre-incubation was performed in pooled HLM (0.2 mg/mL) added with BBR, THB, TFD, or DMB, or rhCYP2D6 (10 pmol/mL) added with BBR, with or without NADPH (1 mM) in phosphate buffer (0.1 M; pH 7.4) for 30 min. Then, the CYP2D6 substrate (5 µM of dextromethorphan) and NADPH (1 mM) were added to the mixture and incubated for another 10 min. All incubations were carried out at 37 °C in a shaking water bath (100 rpm) using eight-well tube strips placed in an 8 × 12 rack, where the final volume of each reaction mixture was 200 µL. Verapamil was used as a positive control. The enzyme reactions were quenched by adding an aliquot (200 µL) of ice-cold ACN containing CBZ (100 nM) as an IS. The samples were centrifuged, and the concentration of CYP2D6 metabolite (i.e., dextrorphan, produced by *O*-demethylation of dextromethorphan) in supernatants were analyzed using the LC-MS/MS method described in Section 2.2.

### 2.4. Determination of Kinetic Parameters for MDI of CYP2D6 by BBR

The metabolism-dependent inhibition of CYP2D6 by BBR was further investigated using the in vitro model mentioned in Section 2.3. BBR (0–16 μM) was incubated with pooled HLM (0.2 mg/mL), NADPH (1 mM), and dextromethorphan (5 µM) in phosphate buffer (0.1 M; pH 7.4) for various incubation time periods (0–30 min). The apparent inactivation rate constants (*k_obs_*) at various BBR concentrations were estimated by fitting first-order kinetics to the relative enzyme activity vs. incubation time profiles. The rate of enzyme inactivation (*k_inact_*) and the inhibition constant (*K_I_*) were determined by fitting the following Equation (1) to the *k_obs_* values at various inhibitor concentrations Equation (1) [31]:

(1) $$\frac{1}{k_{obs}}=\frac{K_{I}}{k_{inact}}\times \frac{1}{\left[I\right]}+\frac{1}{k_{inact}}$$

### 2.5. Reversibility of CYP2D6 Inhibition by BBR

The reversibility of CYP2D6 inhibition by BBR was investigated by ultracentrifugation and potassium ferricyanide (K_3_Fe(CN)_6_) treatment [7]. Ketoconazole (1 µM), diltiazem (100 µM), and mifepristone (30 µM) were used as positive controls for the reversible, quasi-irreversible and irreversible inhibition, respectively. The pre-incubation mixtures consisting of potassium phosphate buffer (0.1 M; pH 7.4), HLM (1 mg/mL), NADPH (1 mM), and inhibitor (total volumes of 180 µL per well) were incubated at 37 °C for 30 min. To verify the reversibility of quasi-irreversible inhibition, potassium ferricyanide (2 mM) was co-incubated with the mixture for 10 min at 37 °C, from which microsomes were re-isolated by ultracentrifugation at 100,000 g for 60 min at 4 °C. The resulting microsomal pellet was washed twice with potassium phosphate buffer (0.1 M; pH 7.4). The obtained microsomes (adjusted to 0.2 mg/mL) were incubated with CYP substrates (25 µM of dextromethorphan for CYP2D6 and 25 µM of MDZ for CYP3A4) and NADPH (1 mM) in potassium phosphate buffer (0.1 M; pH 7.4) for 5 min to measure the CYP activity. The reactions were terminated by the addition of ACN (200 µL) containing CBZ (100 nM). The samples were centrifuged, and the supernatants were analyzed by the abovementioned LC-MS/MS method in Section 2.2.

### 2.6. Metabolic Stability and Metabolite Identification of BBR in Pooled HLM

To evaluate NADPH-dependent metabolism, BBR (1 µM) was incubated with pooled HLM (1 mg/mL) in the presence of the NADPH-regenerating system (1 mM of NADP^+^, 5 mM of G6P, and 1 U/mL of G6PDH) to be a final volume of 200 µL. The reactions were quenched by adding the same volume of ice-cold ACN containing CBZ (100 nM) at 0, 7.5, 15, 30, 60, and 120 min of incubation. The incubation mixtures were centrifuged at 1000× *g* for 20 min at 4 °C, and the supernatants were analyzed by the LC-MS/MS system described in Section 2.2. The sample injection volume was 5 µL, and the separation was performed on an XTerra^®^MS C18 column (2.1 × 150 mm, 5 µm; Waters, Milford, MA, USA). The mobile phases consisted of DDW containing 0.1% (*v*/*v*) of formic acid (A) and methanol (B). A linear gradient elution program of the two mobile phases was performed at a flow rate of 0.2 mL/min, where the changes in the percentage of mobile phase B over time are as follows: 0% at 0 min, 1% at 0.1 min, 48% at 15–17 min, and 100% at 18–24 min, followed by re-equilibration for 3 min. The ionization source settings were optimized to be the ionization voltage of 4500 V, ion source temperature of 550 °C, nebulizing gas flow of 50 L/min, auxiliary gas flow of 40 L/min, and curtain gas flow of 30 L/min.

Meanwhile, to identify the known metabolites of BBR, an aliquot (5 µL) of the supernatant was injected into an accurate-MS system consisting of an Agilent 6530 Q-TOF LC/MS equipped with dual AJS ESI ion source (Agilent Technologies) and Agilent 1200 series HPLC system (Agilent Technologies). Chromatographic separations were performed on an Agilent Eclipse Plus C18 column (150 × 2.1 mm, 3.5 µm; Agilent Technologies) by gradient elution with a binary mobile phase consisting of DDW with 0.1% (*v*/*v*) formic acid (A) and ACN with 0.1% (*v*/*v*) formic acid (B) at a flow rate of 0.2 mL/min, where the mobile phase B was set as follows: 2% at 0 min, 20% at 15 min, and 30% at 20–25 min, followed by re-equilibration for 3 min.

### 2.7. BBR-Nebivolol Interaction in Pooled HLM

The HDI between BBR and nebivolol was assessed using pooled HLM, where nebivolol (1 μM) was incubated with HLM (1 mg/mL) and NADPH-generating system (see Section 2.6) in phosphate buffer (0.1 M; pH 7.4) with BBR at various concentrations (0, 1, 3, and 10 μM). The mixture (total volume of 200 µL) was incubated at 37 °C for 60 min in a shaking water bath (100 rpm), following a 5 min of pre-incubation at 37 °C. The enzyme reaction was quenched by adding ice-cold ACN (200 µL) containing CBZ (100 nM), and the mixture was centrifuged at 1000× *g* for 20 min at 4 °C. The supernatants were analyzed using the same LC-MS/MS method described in Section 2.2, except that a Shimadzu 20AD-XR HPLC system (Shimadzu, Kyoto, Japan) was employed for the chromatographic separation of analytes. The mass transitions of nebivolol and CBZ were *m*/*z* 406 to 151 and *m*/*z* 237 to 194, respectively.

### 2.8. Data Analysis

In vitro metabolic stability parameters, including half-life (*t*_1/2_) and microsomal intrinsic clearance (*CL_int,mic_*), were determined according to our previous report [32]. The degradation *t*_1/2_ values of the microsomal stability studies were calculated using the following Equation (2):

(2) $$t_{1/2}=\frac{0.693}{k},$$

where *k* represents the first-order degradation rate constant. The *k* values were estimated by fitting a one-phase exponential decay model to the parent drug remaining (%) vs. incubation time plots using GraphPad Prism 5.0 (GraphPad Software Inc., San Diego, CA, USA).

The *CL_int,mic_* was determined using the in vitro half-life approach. The *CL_int,mic_* was normalized by the concentration of microsomal protein used (mg/μL), as displayed in the following Equation (3):

(3) $$CL_{int,mic}=\frac{0.693}{t_{1/2}}\cdot \frac{{\left[V\right]}_{incubation\;}\left(µL\right)}{{\left[P\right]}_{incubation\;}\left(\mathrm{mg}\right)}\Rightarrow \left(µL/\min/\mathrm{mg}\;\mathrm{protein}\right)$$

where [*V*] represents the incubation volume (µL), and [*P*] is the amount (mg) of microsomal protein in the incubation mixture.

## 3. Results

### 3.1. CYP Inhibition by BBR and THB

**Figure 1:**
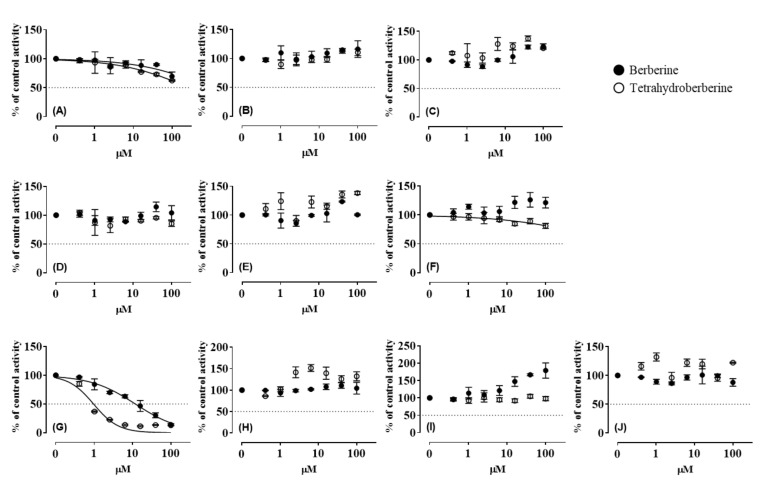
Effects of berberine and tetrahydroberberine on CYP1A2 (**A**), CYP2A6 (**B**), CYP2B6 (**C**), CYP2C8 (**D**), CYP2C9 (**E**), CYP2C19 (**F**), CYP2D6 (**G**), CYP2E1 (**H**), CYP3A4 ((**I**), MDZ), and CYP3A4 ((**J**), TST) in pooled HLM. The activity is expressed as the percentage of control samples containing no inhibitor (100%). Data show the mean ± standard deviation (SD) of three separate samples.

Inhibitory effects of BBR and THB on CYP enzymes were determined in HLM (Figure 1). Ketoconazole used as a positive control for CYP3A4 inhibition showed strong inhibitory activity with an IC_50_ (0.2–0.4 µM) within the reference values [29,33]. BBR displayed inhibitory activity against CYP2D6 (IC_50_ values: 11.9 µM; 95% confidence intervals [CI]: 9.8–14.3 µM). THB exhibited more potent inhibitory activity against CYP2D6 (IC_50_ values: 0.97 µM; 95% CI: 0.76–1.23 µM) than BBR. The other CYP enzymes, including CYP1A2, 2A6, 2B6, 2C8, 2C9, 2C19, 2E1, and 3A4, were not markedly inhibited by BBR or THB. This selective inhibitory effect of both BBR and THB on CYP2D6 could be attributed to their structural similarity.

**Figure 2:**
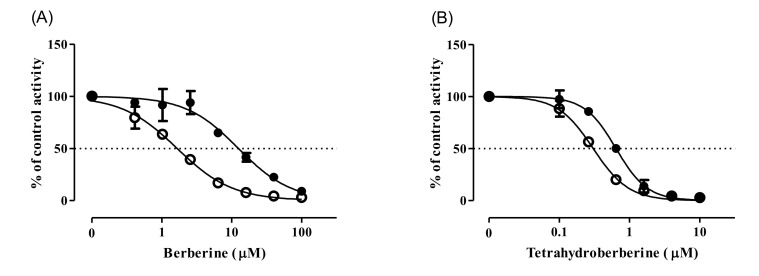
Changes in the inhibition curves of CYP2D6 by BBR (**A**) and THB (**B**) after pre-incubation of pooled HLM with (empty circle, ○) or without (solid circle, ●) NADPH for 30 min. The activity is expressed as the percentage of control samples containing no inhibitor (100%). Data show the mean ± SD of three separate samples.

To investigate the mechanism of CYP2D6 inhibition by BBR and THB, their MDI potentials were demonstrated by assessing the IC_50_ shift after pre-incubation with pooled HLM and NADPH [7]. Verapamil was employed as a positive control for the MDI of CYP3A4 (substrate: MDZ), which showed more than a 7.1-fold reduction in IC_50_ values (from 13.3 µM to 1.87 µM) after the pre-incubation with HLM and NADPH. As expected from our previous report [28], the IC_50_ value of BBR against CYP2D6 decreased from 12.4 µM (95% CI: 10.3–15.0 µM) to 1.66 µM (95% CI: 1.48–1.85 µM) after the pre-incubation, implying the presence of MDI (Figure 2A). Also, in rhCYP2D6, pre-incubation of BBR with NADPH resulted in a 3.1-fold reduction in IC_50_ values (1.73 µM; 95% CI: 1.44–2.07 µM) compared to those obtained in the absence of NADPH (5.31 µM; 95% CI: 4.55–6.20 µM) (Supplementary Materials, Figure S1). Similarly, the pre-incubation of THB with NADPH decreased IC_50_ value against CYP2D6 from 0.661 µM (95% CI: 0.612–0.713 µM) to 0.306 µM (95% CI; 0.279–0.335 µM) (Figure 2B).

### 3.2. Metabolic Stability and Metabolite Identification of BBR in HLM

The metabolic stability of BBR was evaluated using HLM, where the estimated *t*_1/2_ of BBR was 35.4 min (Supplementary Materials, Figure S2). *CL_int,mic_* of BBR was calculated to be 7.82 µL/min/mg protein, suggesting that BBR is moderately stable in HLM.

The formation of BBR metabolites was monitored over the 120 min of incubation with HLM (Table 1), in which the identification and quantification of metabolites were performed simultaneously using authentic standards, including BRB, TFD, DMB, and JTZ. As the incubation time elapsed, the concentrations of TFD, DMB, and M1 (an unknown metabolite observed at *m/z* 310) in the reaction mixture increased gradually. BRB exhibited a slight increase in its concentration, whereas JTZ showed no difference. M1 was further characterized using an accurate-MS system (Supplementary Materials, Table S2). The proposed chemical formula of M1 is C_18_H_16_NO_4_^+^ with a mass error of 1.9 ppm, and the fragment ions of M1 were observed at *m/z* 295.0824 and 267.0890. Notably, M1 was also generated from the incubation of TFD or DMB with HLM in the presence of NADPH, but not in the absence of NADPH (Supplementary Materials, Table S3), suggesting that M1 could be produced from BBR via TFD or DMB. The product ions of M1 were observed at *m/z* 295 and 267 (Supplementary Materials, Table S2), showing a difference of 12 mass units compared to the product ions of TFD (*m/z* 307 and 279) and a difference of 14 mass units compared to the product ions of DMB (*m/z* 309). These results suggest that M1 could be demethylene-TFD.

### 3.3. CYP Inhibition by BRB, TFD, DMB, and JTZ

The effects of BRB, TFD, DMB, and JTZ on CYP activities were evaluated using HLM (Table 2 and Figures S3–S7 in Supplementary Materials). BRB exhibited pan-CYP inhibitory activities with IC_50_ values ranging from 17 to 96 µM. Similarly, TRB, an analogue of BRB, also showed pan-CYP inhibitory activities with much lower IC_50_ values ranging from 2.7 to 9.6 µM, suggesting that TRB is a more potent pan-CYP inhibitor than BRB. TFD, DMB, and JTZ showed inhibitory activities against CYP2D6 and CYP2E1. JTZ also exhibited inhibitory effects on CYP2C8. DMB and JTZ strongly inhibited the metabolism of TST, a putative substrate of CYP3A4. However, the metabolism of MDZ, another substrate of CYP3A4, was inhibited only by DMB with a much higher IC_50_ value.

**Figure 3:**
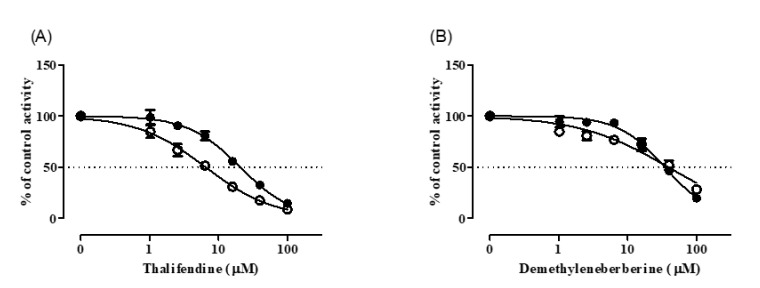
Changes in the inhibition curves of CYP2D6 by TFD (**A**) and DMB (**B**) after pre-incubation of pooled HLM with (empty circle, ○) or without (solid circle, ●) NADPH for 30 min. The activity is expressed as the percentage of control samples containing no inhibitor (100%). Data show the mean ± SD of three separate samples.

The contribution of TFD and DMB (i.e., BBR metabolites) on the BBR-mediated MDI of CYP2D6 was assessed by monitoring their IC_50_ shifts after pre-incubation with pooled HLM and NADPH (Figure 3). Pre-incubation of TFD in the presence of NADPH resulted in a 3.2-fold reduction in IC_50_ values (6.49 µM; 95% CI: 5.8–7.2 µM) compared to those obtained in the absence of NADPH (20.6 µM; 95% CI: 18.9–22.5 µM) (Figure 3A). In contrast, the IC_50_ value of DMB increased after pre-incubation with NDAPH from 34.7 µM (95% CI: 31.4–38.3 µM) to 54.9 µM (95% CI: 48.0–62.9 µM) (Figure 3B).

### 3.4. Inhibition Kinetics of CYP2D6 by BBR

**Figure 4:**
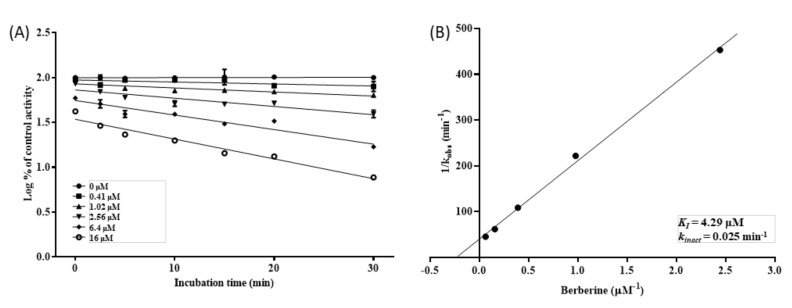
Time-dependent inhibition of CYP2D6 by BBR. (**A**) BBR was incubated for 0, 1, 5, 10, 15, or 30 min with NADPH in pooled HLM. CYP2D6 activity is expressed as the log percentage of control group containing no inhibitor at zero time. Data show the mean ± SD for three separate samples. (**B**) Double-reciprocal plot of *k_obs_* and BBR concentrations was plotted to determine *K_I_* and *k_inact_* for the inactivation of CYP2D6 by BBR.

The inhibition kinetics of BBR against CYP2D6 were characterized by determining *k_inact_* and *K_I_* using the relationship between *k_obs_* and inhibitor concentrations (Figure 4). The estimated values of *k_inact_*, *K_I_* and *k_inact_*/*K_I_* of BBR against CYP2D6 were 0.025 min^−1^, 4.29 µM and 5.83 mL/min/µmol, respectively.

**Figure 5:**
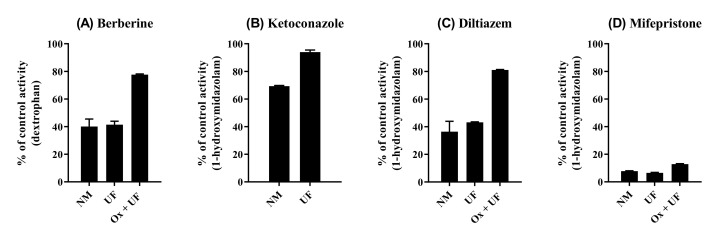
Reversibility of CYP inhibition by BBR (**A**), ketoconazole ((**B**)—a reversible inhibitor for CYP3A4), diltiazem ((**C**)—a quasi-irreversible inhibitor for CYP3A4), and mifepristone ((**D**)—an irreversible inhibitor for CYP3A4). BBR (5 μM), diltiazem (100 μM), or mifepristone (30 μM) was incubated for 30 min with NADPH in HLM, and microsomal protein was re-isolated by ultracentrifugation with (Ox + UF) or without (UF) CYP oxidation. Ketoconazole (1 μM) was incubated for 30 min without NADPH in HLM, and microsomal protein was re-isolated by ultracentrifugation (UF). The activity of re-isolated microsomal protein was compared with that with no manipulation (NM). Activity is expressed as the percentage of control samples containing no inhibitor (100%). Data show the mean ± SD for three separate samples.

The mode of CYP2D6 inhibition was also investigated, where the reversibility of CYP inhibition was evaluated via the re-isolation of HLM using ultracentrifugation coupled with CYP oxidation (Figure 5). As expected from the MDI observed in the pre-incubation study (Figure 2A), the inhibition of CYP2D6 by BBR was not reversed by a simple dilution (i.e., ultracentrifugation), whereas CYP3A4 inhibition by ketoconazole (reversible inhibitor) exhibited almost complete reversal (≈95%) after ultracentrifugation. Noteworthy is that BBR-treated HLM recovered nearly 80% of its activity by adding the CYP oxidation step (i.e., potassium ferricyanide treatment) before ultracentrifugation, displaying a similar pattern with diltiazem (quasi-irreversible inhibitor). The inhibition by mifepristone (irreversible inhibitor) was reversed by neither ultracentrifugation nor by CYP oxidation.

### 3.5. Metabolic Interaction between BBR and Nebivolol in HLM

**Figure 6:**
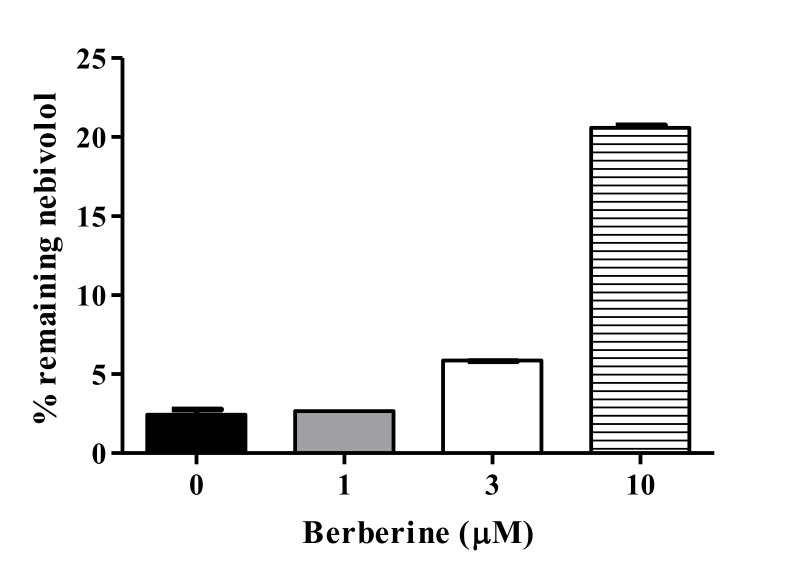
Effects of BBR on nebivolol metabolism in pooled HLM. Nebivolol (1 μM) was incubated with pooled HLM (1 mg/mL) in the presence or absence of BBR at various concentrations for 60 min. Data are presented as the mean ± SD of three separate samples.

The potential of interaction between BBR and possible concomitant medication was investigated by assessing the effects of BBR on microsomal metabolism of nebivolol, a β-blocker used to treat high blood pressure and heart failure (Figure 6). Metabolic clearance of nebivolol is governed by CYP2D6, although CYP2C19 and CYP3A4 are also engaged to a lesser extent [34]. Microsomal clearance of nebivolol at a concentration of 1 µM was significantly inhibited by BBR in a concentration-dependent manner. The metabolic clearance of nebivolol in HLM treated with 10 μM BBR may be mediated by CYP2C19 and/or CYP3A4 that are not inhibited by BBR.

## 4. Discussion

CYP inhibition is one of the most common mechanisms for causing clinically relevant drug–drug interactions, and it is important to study the mechanism of CYP inhibition. A previous study showed that BBR possesses inhibitory activity against CYP2D6 [35]. In our previous study, H-Tang, containing BBR as a main active ingredient, inhibited CYP2D6 in a quasi-irreversible manner [28]. In addition, the MDI of CYP2D6 by BBR was also elucidated. However, no information of a detailed mechanism of BBR-induced CYP2D6 inhibition has been reported, encouraging the present study aiming to evaluate the inhibition mode of BBR against CYP enzymes using HLM.

Our data reveals that BBR exhibits selective inhibition of CYP2D6 with 11.9 µM of IC_50_ observed in direct CYP inhibition assay. And the pre-incubation of HLM with BBR to produce its metabolites resulted in an approximately 7.5-fold decrease in IC_50_, indicating that the inhibition of CYP2D6 by BBR is time-dependent inhibition or MDI. Moreover, THB, an analogue of BBR, showed MDI of CYP2D6 with lower IC_50_ values (0.661 µM of IC_50_ without NADPH and 0.306 µM of IC_50_ with NADPH), where the shift in IC_50_ (2.2-fold) was also smaller, compared to BBR. In the kinetic studies of CYP2D6 inhibition, values of *k_inact_*, *K_I_*, and the ratio of *k_inact_* to *K_I_* of BBR against CYP2D6 were 0.025 min^−1^, 4.29 µM, and 5.83 mL/min/µmol, respectively. The value of k_inact_/K_I_ of BBR against CYP2D6 was comparable to that of diltiazem or verapamil [36,37], both of which are frequently used as positive controls for MDI.

The metabolic characterization of BBR using HLM showed that BBR exhibited moderate metabolic stability with 35.4 min of *t*_1/2_ and 7.82 µL/min/mg protein of *CL_int,mic_*. The metabolite identification studies using QTRAP LC-MS/MS and Q-TOF LC/MS with authentic standards showed that BBR was metabolized to TFD, DMB, and M1 (*m/z* 310), and to lesser extent BRB but not JTZ. TFD and DMB, but not BRB or JTZ, were identified as metabolites of BBR in a previous study using HLM and recombinant human CYP, although all four metabolites were observed in rat liver [27]. M1, found in this study for the first time, was produced in HLM incubated with TFD and DMB, and the proposed chemical formula of M1 is C_18_H_16_NO_4_^+^. The product ions of M1 and TFD were observed at *m/z* 295 and 267, and 307 and 279, respectively (Supplementary Materials, Table S2), displaying a difference of 12 mass unit between the product ions of M1 and TFD. This result suggests that M1 could be demethylene-TFD. Proposed metabolic pathways of BBR are presented in Supplementary Materials (Figure S8).

In the direct CYP inhibition assay, BBR analogues, including BRB, TFD, DMB, and JTZ, exhibited CYP2D6 inhibition activities. However, these compounds are less selective to CYP2D6 than BBR, showing pan-CYP inhibition for BRB and CYP2E1/3A4 inhibition for TFD, DMB, and JTZ. According to IC_50_ value, JTZ inhibited CYP2D6 to a comparable degree with BBR, whereas BRB, TFD, and DMB were less potent than BBR. In addition, all CYPs tested in this study were also inhibited by TRB, an analogue of BRB. These results suggest that minor structural modifications of BBR lead to significant changes in CYP inhibitory properties of BBR and its analogues.

The MDI study of CYP2D6 was performed using TFD and DMB, which were found to be major metabolites of BBR via Q-TOF LC/MS analysis. Notably, TFD resulted in a significant IC_50_ shift (3.2-fold) after pre-incubation with HLM, implying MDI of CYP2D6 by BBR can be attributed to this metabolite. However, the IC_50_ value of TFD against CYP2D6 obtained from the pre-incubation study was higher than that of BBR, which suggests that unidentified metabolite(s) of BBR may be responsible for metabolism-dependent CYP2D6 inhibition of BBR.

In the reversibility study, the CYP2D6 inhibition by BBR was restored by ultracentrifugation coupled with CYP oxidation, but not ultracentrifugation only, suggesting that inhibitory mode of BBR against CYP2D6 may be quasi-irreversible. Irreversible and quasi-reversible inhibitions are collectively expressed as mechanism-based inhibition (MBI), which involves the generation of reactive intermediates from inhibitors by metabolic enzymes, especially CYPs [38]. In the irreversible inhibition, the reactive intermediate modifies the CYP enzymes forming a covalent bond, resulting in an irreversible loss of enzyme activity [9]. The quasi-irreversible inhibition results from the formation of a stable MIC with the ferrous form of the heme iron atom. In the present study, BBR, THB, and TFD but not DMB exhibited MDI of CYP2D6. These results raise the possibility that methylenedioxybenzene moiety of BBR may play a critical role in the quasi-irreversible inhibition by BBR, which is supported by previous results showing the role of methylenedioxybenzenes in the formation of MICs [39,40]. Moreover, it has been suggested that the formation of the highly reactive methylenedioxy carbene from methylenedioxybenzene can form a MIC [41]. In the present study, BBR metabolite(s) that is responsible for the quasi-irreversible inhibition of CYP2D6 was not identified in HLM incubated with BBR, which may be attributed to reactivity or instability of metabolite intermediates forming MIC.

H-Tang containing BBR as the main component is one of the most frequently used traditional herbal medicines to treat inflammation, hypertension, and gastrointestinal disorders [28]. Recently, it has been suggested that BBR could serve as an alternative to antihyperlipidemic drugs for patients with statin resistance [24]. Thus, frequent use of BBR may lead to HDIs through CYP2D6 inhibition. The present study showed that the microsomal clearance of nebivolol was inhibited by BBR in a concentration-dependent manner, suggesting a careful evaluation on the use of BBR concomitantly with drugs metabolized by CYP2D6. In clinical PK studies using a single administration of BBR, the maximum plasma concentration was highly variable (from less than 1 nM to more than 1 µM) due to low bioavailability and variations in gut microbiota [42,43,44]. A quasi-irreversible inhibitor may inactivate its own clearance mechanism, resulting in reduced clearance and accumulation with repeated administration [39]. The present results warrant a further evaluation of BBR–drug interaction in clinical situations and a determination of inter-individual variability in CYP2D6 inhibition by BBR, based on genetic polymorphism of CYP2D6.

## 5. Conclusions

BBR exhibits selective quasi-irreversible inhibition of CYP2D6 with 0.025 min^−1^ of *k_inact_*, 4.29 µM of *K_I_*, and 5.83 mL/min/µmol of *k_inact_*/*K_I_*. In pooled HLM, BBR showed moderate metabolic stability with 35.4 min of *t*_1/2_ and 7.82 µL/min/mg protein of *CL_int,mic_*, being metabolized to TFD, DMB, and M1 (at *m/z* 310, proposed as demethylene-TFD), and to lesser extent BRB. TFD, DMB, and BRB were not selective inhibitors of CYP2D6 with less potency than BBR, based on a comparison of IC_50_ values. BBR and TFD, but not DMB, exhibited MDI, suggesting that methylenedioxybenzene moiety of BBR may be crucial for its quasi-irreversible inhibition of CYP2D6. Considering repeated administration of a quasi-irreversible inhibitor can lead to its accumulation through inactivation of its own metabolism mechanism, BBR–drug interactions need to be further investigated in clinical situations.

## Figures and Tables

**Table 1:** Elimination of BBR and formation of its metabolites in pooled HLM.

Time (min)	BBR (nM)	BRB (nM)	TFD (nM)	DMB (nM)	JTZ (nM)	M1 (*m/z* = 310) (1000 × cps)
0	1049 ± 16	5.4 ± 0.1	5.4 ± 0.1	6.1 ± 0.1	10.4 ± 0.3	1.3 ± 0.25
7.5	807 ± 11	5.6 ± 0.0	20.9 ± 0.4	24.1 ± 0.3	10.7 ± 0.1	28 ± 0.66
15	713 ± 12	5.8 ± 0.1	33.3 ± 0.6	38.1 ± 0.5	10.9 ± 0.3	80 ± 0.31
30	539 ± 4	6.0 ± 0.1	44.3 ± 1.0	52.6 ± 1.9	10.5 ± 0.3	220 ± 2.6
60	313 ± 15	6.1 ± 0.0	50.4 ± 0.7	64.3 ± 1.6	10.0 ± 0.2	540 ± 15
120	147 ± 5	6.2 ± 0.0	49.3 ± 1.0	75.0 ± 1.0	9.6 ± 0.2	1100 ± 26

Berberine (1 μM) was incubated for 0, 7.5, 15, 30, 60, or 120 min in pooled HLM in the presence of NADPH-generating system. Each value represents the mean ± SD for three separate samples.

**Table 2:** IC_50_ values of BRB, TFD, DMB, JTZ and TRB against CYP enzymes in pooled HLM.

IC_50_ (µM)	CYP Enzyme									
	1A2	2A6	2B6	2C8	2C9	2C19	2D6	2E1	3A4 (MDZ)	3A4 (TST)
**BRB**	51 (41–62)	17 (14–22)	96 (79–116)	72 (62–83)	57 (52–64)	49 (41–59)	45 (41–49)	34 (29–41)	28 (26–31)	45 (38–53)
**TFD**	>100	>100	>100	>100	>100	>100	28 (24–33)	32 (25–42)	>100	>100
**DMB**	>100	>100	>100	>100	>100	>100	32 (27–37)	7.7 (6.0–9.8)	66 (55–78)	7.3 (5.6–9.4)
**JTZ**	>100	>100	>100	26 (19–37)	>100	>100	10 (8–12)	11 (10–13)	>100	11 (10–13)
**TRB**	6.9 (6.5–7.4)	4.7 (4.1–5.3)	9.6 (8.7–10.5)	9.5 (7.9–11.3)	6.5 (5.8–7.3)	5.4 (5.0–5.8)	3.4 (3.2–3.7)	2.7 (2.5–2.9)	5.3 (4.7–6.1)	4.6 (4.1–5.2)

Each compound was incubated at 0, 0.41, 1.02, 2.56, 6.4, 16, 40 or 100 μM in pooled HLM. Value given in parentheses represent 95% confidence intervals.

## Supplementary Materials

The following are available online at https://www.mdpi.com/1999-4923/12/10/916/s1, Table S1: MRM parameters for mass spectrometric detection of analytes using API4000 Q-TRAP system; Table S2: Putative metabolites of BBR identified in pooled HLM using 6530 Q-TOF LC-MS/MS; Table S3: Putative M1 metabolite produced in pooled HLM incubated with THD or DMB; Figure S1: Changes in the inhibition curves of rhCYP2D6 by BBR after pre-incubation with or without NADPH; Figure S2: Metabolic stability of BBR in pooled HLM incubated with NADPH-generating system; Figures S3–S7: Effects of THB, BRB, TFD, DMB, and JTZ, respectively, on CYP1A2, 2A6, 2B6, 2C8, 2C9, 2C19, 2D6, 2E1, and 3A4 in pooled HLM; Figure S8: Proposed metabolic pathways of BBR in human liver microsomes; Figure S9: Chemical structure of TRB and its NMR spectra. Contents regarding the preparation of TRB are also available.
