# Supplementary material for: Quasi-Irreversible Inhibition of CYP2D6 by Berberine
Source: Pharmaceutics. 2020 Sep 24;12(10):916. doi: 10.3390/pharmaceutics12100916 (PMC7600264; doi:10.3390/pharmaceutics12100916)
Supplement: Supplementary file 1 [file pharmaceutics-12-00916-s001.pdf]

pharmaceutics

Supplementary Materials: Quasi-irreversible Inhibition of CYP2D6 by Berberine

Ha Gyeong Kim, Han Sol Lee, Jang Su Jeon, Young Jae Choi, Yeon Jung Choi, So-Yeol Yoo, Eun-yeong Kim, Kiho Lee, InWha Park, MinKyun Na, Han-Jin Park, Seung-Woo Cho, Jong-Hoon Kim, Jae-Young Lee and Sang Kyum Kim

1. Preparation of Tetrahydroberberrubine

1.1. Extraction and Isolation of Tetrahydroberberrubine

The The fruits of *Nandina domestica* (3.7 kg) were extracted three times with MeOH (3L) at room temperature for 7 days to obtain the MeOH extract (360.0 g). After acid-base extraction, an alkaloid containing fraction (NDA, 15.8 g) was separated into 7 fractions (NDA-1 to NDA-7) by MPLC (SNAP Cartridges KP-Sil, 340 g) using a mixed solvent of *n*-Hexane-EtOAc-formic acid (600:10:3, 600:20:5, 600:30:15, 400:30:15, 300:30:15, 200:30:15, and 100% MeOH). Fraction NDA-6 (1.1 g) was purified by C_{18} HPLC eluting with MeCN-H_2O (linear gradient from 10:90 to 70:30) to yield tetrahydroberberrubine (200 mg).

Tetrahydroberberrubine·acetate: green gum; $[\alpha]_D^{20}$ −188 (*c* 0.5, MeOH); ^{1}H NMR (methanol-d_4, 300 MHz) δ_H 6.88 (1H, d, *J* = 8.1 Hz, H-11), 6.87 (1H, s, H-1), 6.73 (1H, d, *J* = 8.1 Hz, H-12), 6.68 (1H, s, H-4), 5.94 (2H, s, -OCH_2O-), 4.53 (1H, d, *J* = 15.8 Hz, H_2-8), 4.18 (1H, dd, *J* = 4.2, 11.7 Hz, H-14), 3.98 (1H, d, *J* = 15.8 Hz, H_2-8), 3.83 (3H, s, -OCH_3), 3.59 (1H, m, H_2-13), 3.52 (1H, m, H_2-6), 3.16 (1H, m, H_2-5), 2.94 (2H, m, H_2-6 and H_2-13), 2.87 (1H, m, H_2-5), 1.96 (acetate); ^{13}C NMR (methanol-d_4, 75 MHz) δ_C 148.7 (C-10), 148.6 (C-3), 147.0 (C-2), 143.9 (C-9), 128.0 (C-14a), 126.8 (C-8a), 126.0 (C-14a), 120.2 (C-12a), 118.0 (C-12), 112.2 (C-11), 109.3 (C-4), 106.5 (C-1), 102.6 (-OCH_2O-), 61.1 (C-14), 56.6 (-OCH_3), 53.6 (C-8), 51.8 (C-6), 34.9 (C-13), 27.9 (C-5), 21.2 (acetate).

1.2. Structure Determination of Tetrahydroberberrubine

The ^{1}H NMR spectroscopic data exhibited resonances assignable to two tetrasubstituted benzene rings (δ_H 6.88, 1H, d, *J* = 8.1 Hz, H-11; 6.87, 1H, s, H-1; 6.73, 1H, d, *J* = 8.1 Hz, H-12; 6.68, 1H, s, H-4), one dioxymethylene (δ_H 5.94, 2H, s), one methoxy group (δ_H 3.83, 3H, s), one methine (δ_H 4.18, 1H, dd, *J* = 4.2, 11.7 Hz, H-14), and four methylenes (δ_H 4.53, 1H, d, *J* = 15.8 Hz, H_2-8; 3.98, 1H, d, *J* = 15.8 Hz, H_2-8; 3.59, 1H, m, H_2-13; 3.52, 1H, m, H_2-6; 3.16, 1H, m, H_2-5; 2.94, 2H, m, H_2-6 and H_2-13; 2.87, 1H, m, H_2-5). Nineteen carbons signals indicating two benzene rings (δ_C 148.7, 148.6, 147.0, 143.9, 128.0, 126.8, 126.0, 120.2, 118.0, 112.2, 109.3, 106.5), one dioxymethylene (δ_C 102.6), one methoxy group (δ_C 56.6), one methine (δ_C 61.1), and four methylenes (δ_C 53.6, 51.8, 34.9, 27.9) were observed in the ^{13}C NMR spectroscopic data. The structure was deduced as tetrahydroberberrubine on the basis of NMR data interpretation. However, the chemical shifts were slightly different from those in the literature [1] due to the presence of acetate (δ_H 1.96 and δ_C 21.2). Thus, the structure was identified to be tetrahydroberberrubine·acetate (Figure S9). The negative value of specific rotation ($[\alpha]_D^{20}$ −188) revealed the absolute configuration of 14*S*.

pharmaceutics

2. Supplementary Tables

Table S1. MRM parameters for mass spectrometric detection of analytes using API4000 Q-TRAP system.

Metabolite	Transition (*m/z*)	Retention time (min)	Declustering potential (mV)	Collision energy (mV)	Mode
Acetaminophen	152→110	2.96	46	23	ESI+
7-Hydroxycoumarin	163→107	3.26	56	31	ESI+
Hydroxybupropion	256→238	3.07	66	17	ESI+
N-Desethylamodiaquine	328→283	2.95	51	30	ESI+
4-Hydroxytolbutamide	287→171	3.36	86	59	ESI+
4-Hydroxymephenytoin	235→150	3.21	60	17	ESI+
Dextrorphan	258→157	3.05	71	51	ESI+
6-Hydroxychlorzoxazone	184→120	3.25	81	-26	ESI-
1-Hydroxymidazolam	342→324	3.23	96	29	ESI+
6β-Hydroxytestosterone	305→269	3.47	81	21	ESI+
Berberrubine	322→307	16.88	36	39	ESI+
Thalifendine	322→307	16.08	46	31	ESI+
Demethyleneberberine	324→309	15.37	41	32	ESI+
Jatrorrhizine	338→323	16.29	46	31	ESI+
Demethylenethalifendine (M1)	310→295	14.24	36	26	ESI+

ESI+: positive electrospray ionization; ESI-: negative electrospray ionization.

Table S2. Putative metabolites of berberine identified in pooled HLM using 6530 Q-TOF LC-MS/MS.

	Compound	Formula	Expected *m/z* ([M+H]+)	Observed *m/z* ([M+H]+)	Mass error (ppm)	t_R (min)	Fragment ions from Q-TOF
**Reference**	Berberine	$C_{20}H_{18}NO_4^+$	336.1230	336.1227	-0.9	20.09	321.0993, 320.0923, 306.0767, 304.0974, 292.0969, 278.0808, 275.0930
	Berberrubine	$C_{19}H_{16}NO_4^+$	322.1074	322.1074	0.0	18.91	307.0845, 279.0888
	Thalifendine	$C_{19}H_{16}NO_4^+$	322.1074	322.1075	0.3	18.05	307.0841, 279.0894
	Demthyleneberberine	$C_{19}H_{18}NO_4^+$	324.1230	324.1217	-4.0	16.06	309.0974, 308.0911, 294.0749, 292.0950, 280.0958, 266.0812, 263.0925
	Jatrorrhizine	$C_{20}H_{20}NO_4^+$	338.1387	338.1383	-1.2	18.35	323.1151, 322.1079, 308.0910, 306.1123, 294.1119, 280.0966, 279.0883, 277.1104
	M1	$C_{18}H_{16}NO_4^+$	310.1074	No reference			No reference
**Incubation for 120 min**	Berberine	$C_{20}H_{18}NO_4^+$	336.1230	336.1235	1.5	20.09	321.0997, 320.0928, 306.0766, 304.0973, 292.0977, 278.0811, 275.0942
	Berberrubine	$C_{19}H_{16}NO_4^+$	322.1074	N.D.	N.A.	N.A.	N.D.
	Thalifendine	$C_{19}H_{16}NO_4^+$	322.1074	322.1074	0.0	18.08	307.0847, 279.0886
	Demthyleneberberine	$C_{19}H_{18}NO_4^+$	324.1230	324.1223	-2.2	16.11	309.0989, 308.0908, 294.0757, 292.0963, 280.0961, 266.0813, 263.0932
	Jatrorrhizine	$C_{20}H_{20}NO_4^+$	338.1387	338.1382	-1.5	18.37	323.1145, 322.1076, 308.0914, 306.1124, 294.1121, 280.0961, 279.0885, 277.1091
	M1	$C_{18}H_{16}NO_4^+$	310.1074	310.1080	1.9	13.38	295.0824, 267.0890

N.A.: not applicable; N.D.: not detected; t_R: retention time.

Table 3. Putative M1 metabolite produced in pooled HLM incubated with thalifendine or demethyleneberberine.

Precursor molecules	Incubation time (min)[a]	M1 signal at *m/z* 310 (10^3 × cps)[b]
Thalifendine	0	6 ± 1
	30	2200 ± 125
Demethyleneberberine	0	107 ± 3
	30	432 ± 27

[a] Thalifendine or demethyleneberberine (1 μM) was incubated with pooled HLM in the presence of NADPH-generating system; [b] Each value represents the mean ± SD for three separate samples.

3. Supplementary Figures

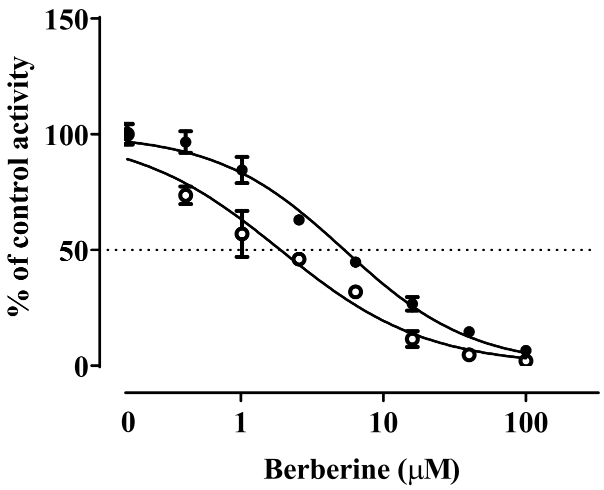

Figure S1. Changes in the inhibition curves of rhCYP2D6 by berberine after pre-incubation with (empty circle, ○) or without (solid circle, ●) NADPH for 30 min. The activity is expressed as the percentage of control samples containing no inhibitor (100%). Data show the mean ± SD of three separate samples.

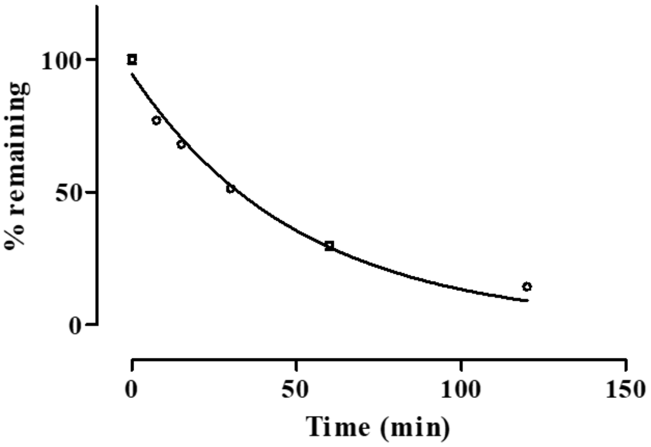

Figure S2. Metabolic stability of berberine in pooled HLM incubated with NADPH-generating system. HLM were incubated with 1 μM berberine for 120 min. Each value represents the mean ± SD for three separate samples.

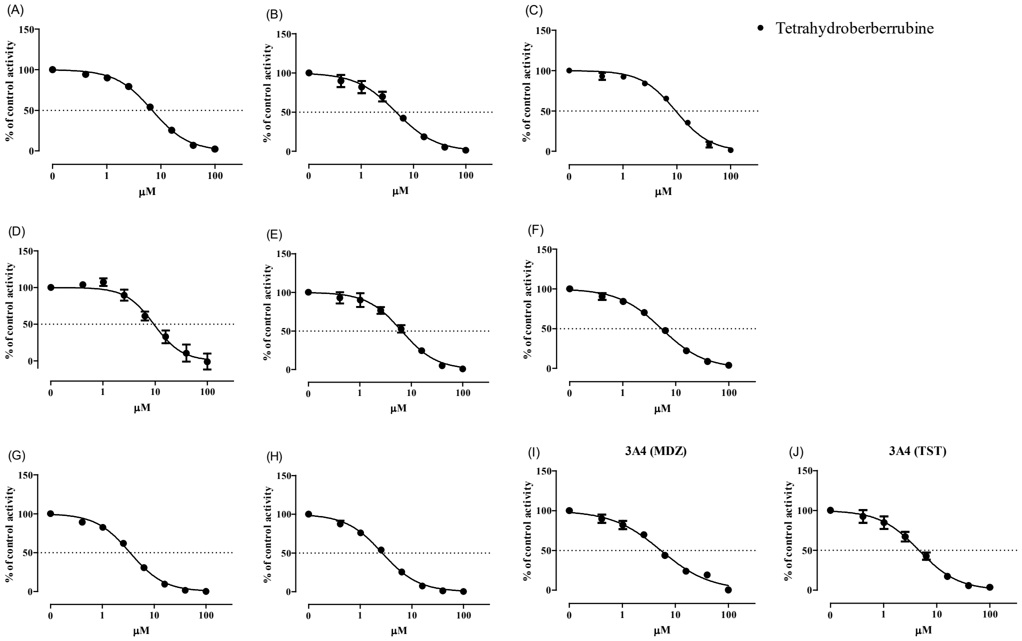

Figure S3. Effects of tetrahydroberberrubine on CYP1A2 (**A**), CYP2A6 (**B**), CYP2B6 (**C**), CYP2C8 (**D**), CYP2C9 (**E**), CYP2C19 (**F**), CYP2D6 (**G**), CYP2E1 (**H**), CYP3A4 (**I**, midazolam), and CYP3A4 (**J**, testosterone) in pooled HLM. The activity is expressed as the percentage of control samples containing no inhibitor (100%) Data show the mean ± SD of three separate samples.

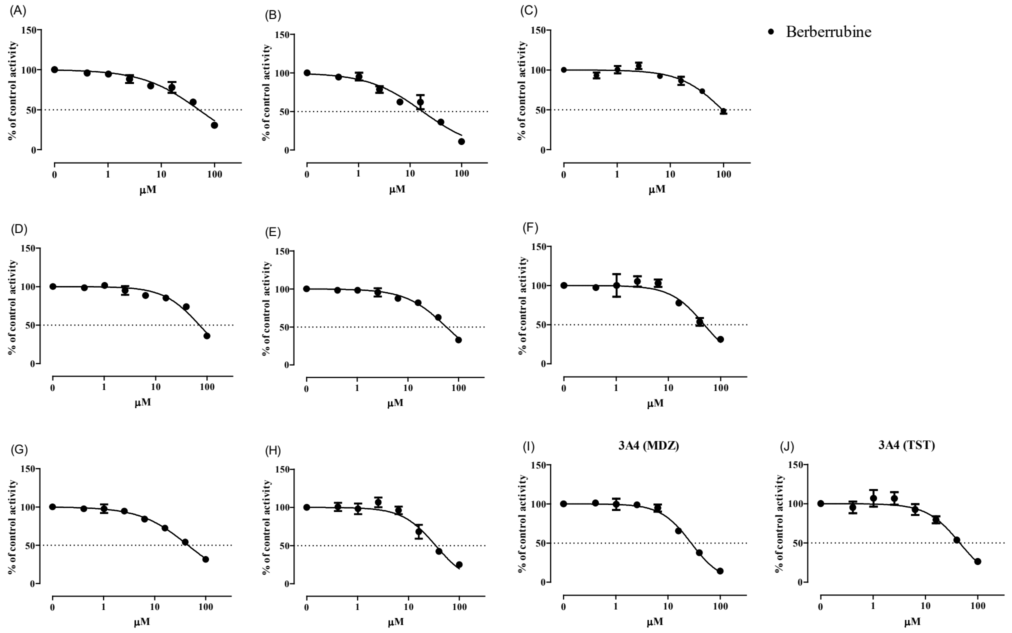

Figure S4. Effects of berberrubine on CYP1A2 (**A**), CYP2A6 (**B**), CYP2B6 (**C**), CYP2C8 (**D**), CYP2C9 (**E**), CYP2C19 (**F**), CYP2D6 (**G**), CYP2E1 (**H**), CYP3A4 (**I**, midazolam), and CYP3A4 (**J**, testosterone) in pooled HLM. The activity is expressed as the percentage of control samples containing no inhibitor (100%) Data show the mean ± SD of three separate samples.

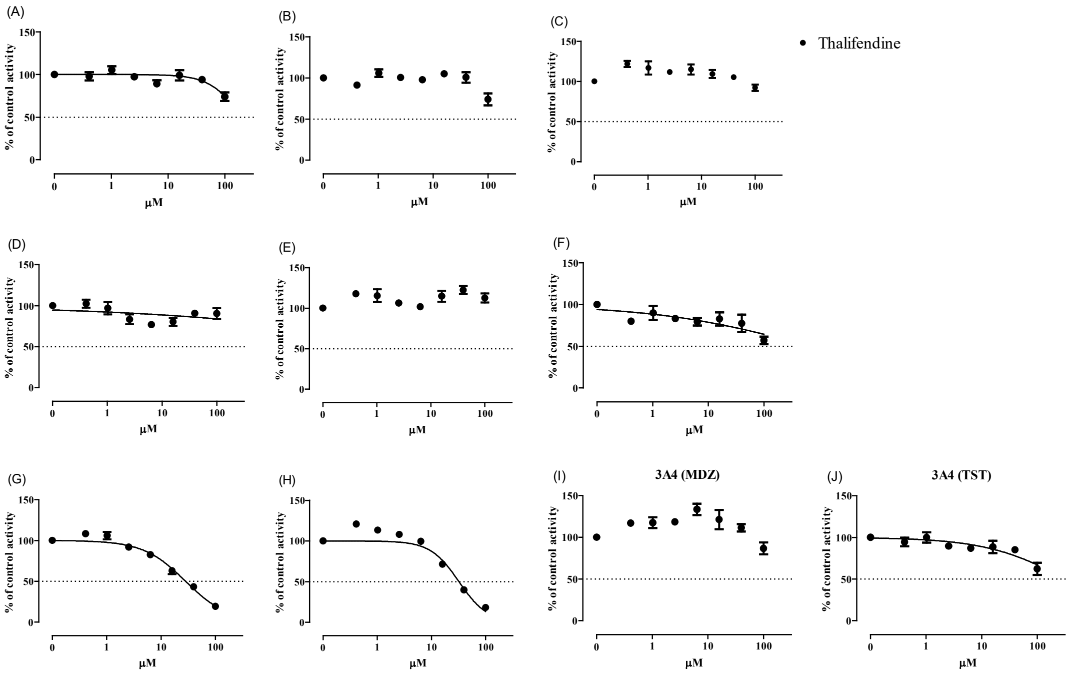

Figure S5. Effects of thalifendine on CYP1A2 (**A**), CYP2A6 (**B**), CYP2B6 (**C**), CYP2C8 (**D**), CYP2C9 (**E**), CYP2C19 (**F**), CYP2D6 (**G**), CYP2E1 (**H**), CYP3A4 (**I**, midazolam), and CYP3A4 (**J**, testosterone) in pooled HLM. The activity is expressed as the percentage of control samples containing no inhibitor (100%) Data show the mean ± SD of three separate samples.

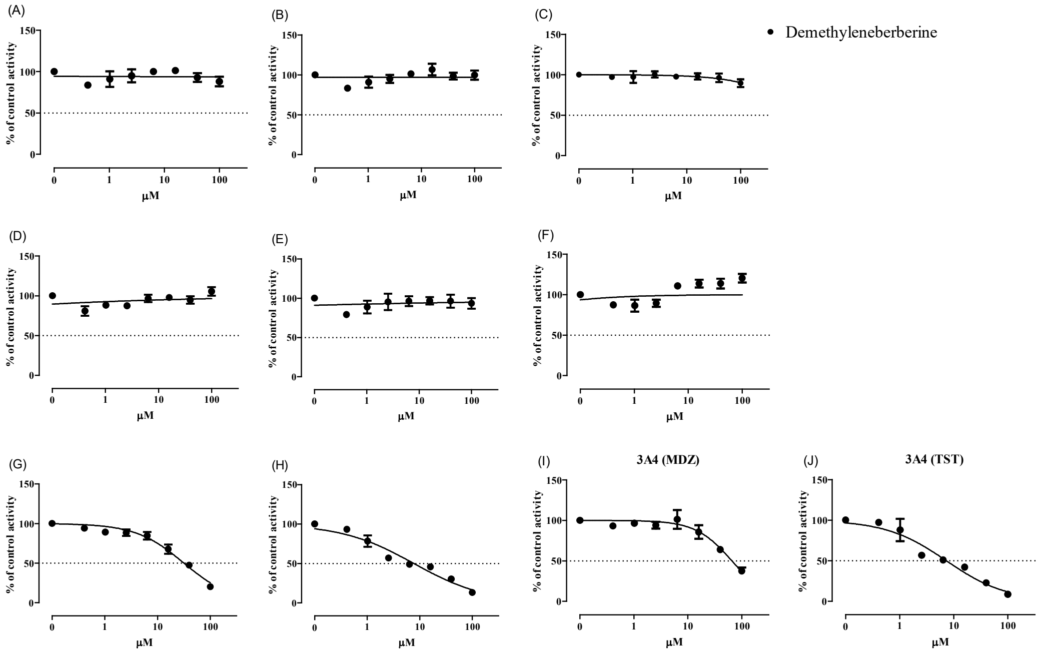

Figure S6. Effects of demethyleneberberine on CYP1A2 (**A**), CYP2A6 (**B**), CYP2B6 (**C**), CYP2C8 (**D**), CYP2C9 (**E**), CYP2C19 (**F**), CYP2D6 (**G**), CYP2E1 (**H**), CYP3A4 (**I**, midazolam), and CYP3A4 (**J**, testosterone) in pooled HLM. The activity is expressed as the percentage of control samples containing no inhibitor (100%) Data show the mean ± SD of three separate samples.

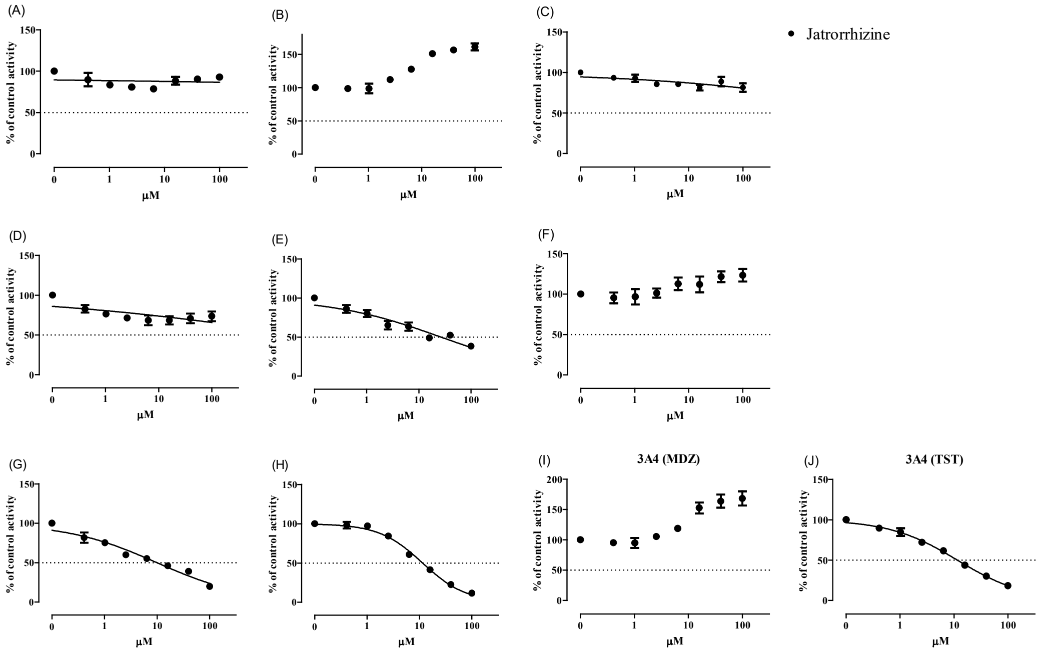

Figure S7. Effects of jatrorrhizine on CYP1A2 (**A**), CYP2A6 (**B**), CYP2B6 (**C**), CYP2C8 (**D**), CYP2C9 (**E**), CYP2C19 (**F**), CYP2D6 (**G**), CYP2E1 (**H**), CYP3A4 (**I**, midazolam), and CYP3A4 (**J**, testosterone) in pooled HLM. The activity is expressed as the percentage of control samples containing no inhibitor (100%) Data show the mean ± SD of three separate samples.

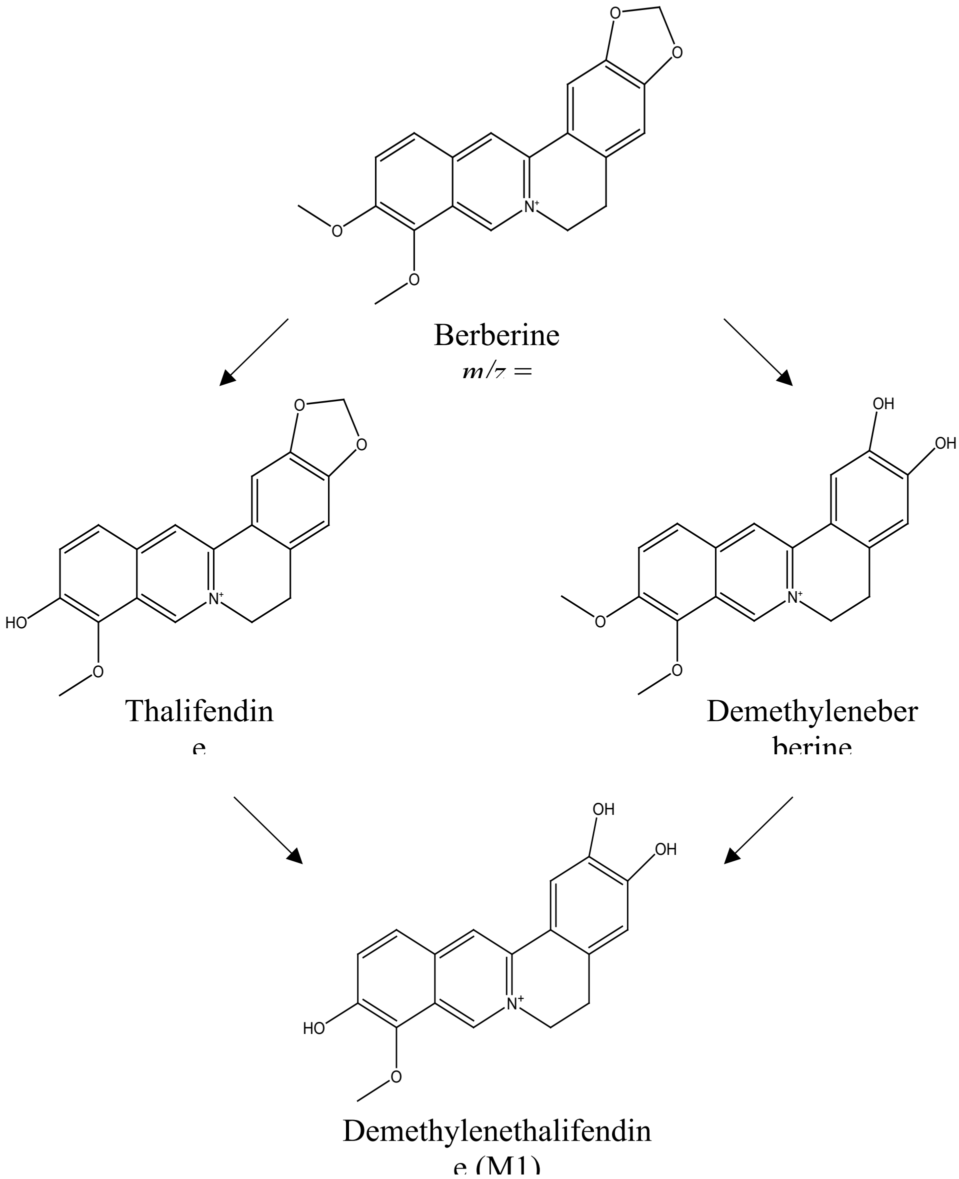

Figure S8. Proposed metabolic pathways of berberine in human liver microsomes.

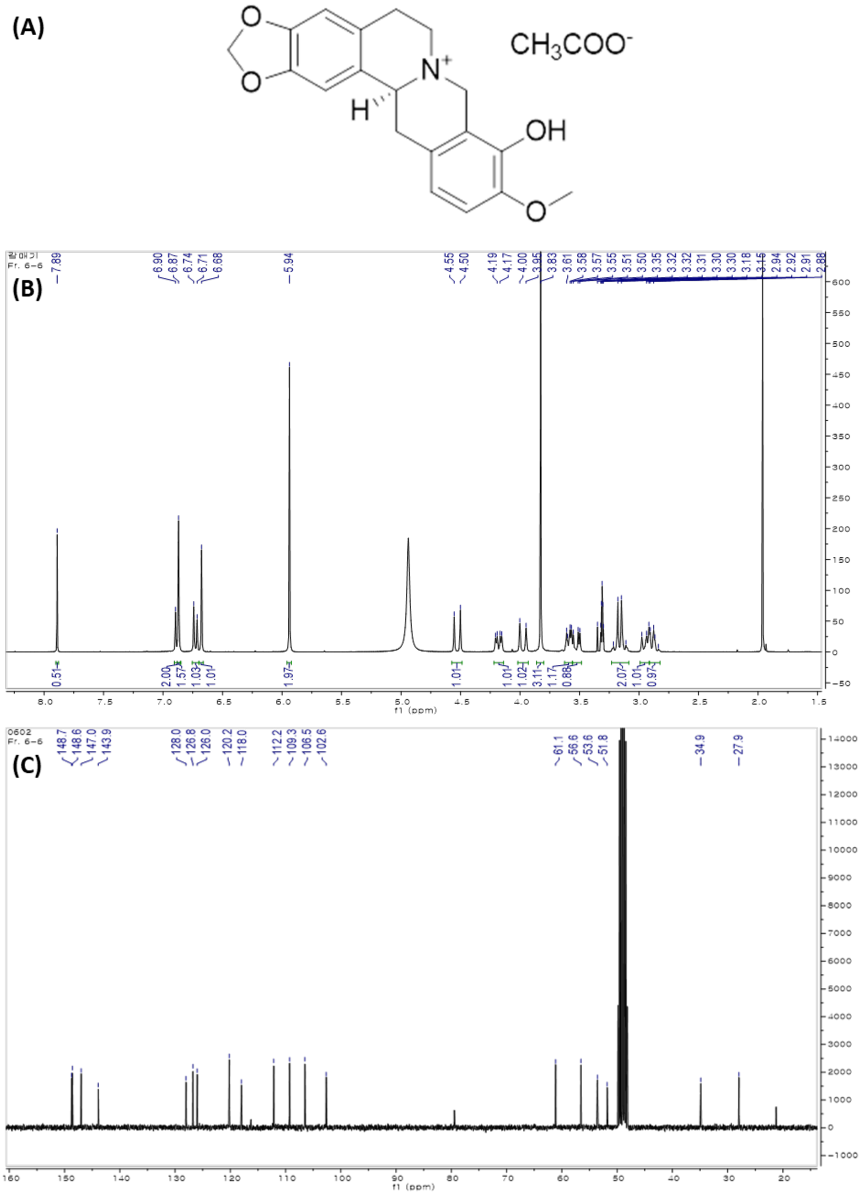

Figure S9. Chemical structure of tetrahydroberberrubine (**A**) The ^{1}H NMR (**B**) and ^{13}C NMR (**C**) spectroscopic data are presented.

4. Reference

1. Ge, H.X.; Zhang, J.; Dong, Y.; Cui, K.; Yu, B.Y. Unique biocatalytic resolution of racemic tetrahydroberberrubine via kinetic glucosylation and enantio-selective sulphation. *Chem. Commun.* **2012**, *48*, 6127–6129.

© 2020 by the authors. Submitted for possible open access publication under the terms and conditions of the Creative Commons Attribution (CC BY) license (http://creativecommons.org/licenses/by/4.0/).
